# Hydralazine-Induced Lupus Syndrome Manifesting as Large Pericardial Effusion

**DOI:** 10.7759/cureus.9867

**Published:** 2020-08-19

**Authors:** Nathaniel R Wilson, Siddharth Chauhan, Gabriel Aisenberg

**Affiliations:** 1 Internal Medicine, University of Texas Health Science Center at Houston, Houston, USA

**Keywords:** hydralazine-induced lupus syndrome, drug-induced lupus (dil), pericardial effusion

## Abstract

Hydralazine-induced lupus syndrome (HILS) is a rare clinical entity with variable manifestations. Pericardial involvement is an uncommon but serious manifestation of the condition. In this report, we present a case of large symptomatic pericardial effusion secondary to HILS. We highlight the important considerations in the evaluation and management of this rare syndrome. HILS should be considered in the differential diagnosis for cardiac tamponade of otherwise unclear etiology in patients taking 100 mg daily or more of hydralazine for longer than three months. A temporal association between the offending drug and presenting symptoms, resolution of symptoms upon discontinuation, and a positive anti-histone antibody test can all support the diagnosis of this syndrome.

## Introduction

Hydralazine, a frequently used blood pressure medication, has been associated with a clinical syndrome similar to idiopathic lupus erythematosus: hydralazine-induced lupus syndrome (HILS). It has an incidence rate of 7-13% and typically presents with fever, myalgias, rash, arthritis, and serositis [1,2]. Pericardial involvement is an uncommon but serious manifestation of this syndrome that must be recognized in patients taking hydralazine [3-8].

## Case presentation

A 71-year-old female with a distant history of renal and breast cancer, stage IIIb chronic kidney disease (estimated glomerular filtration rate of 34 mL/min/1.73m^2^), and longstanding hypertension on hydralazine 100 mg twice daily for two years presented to the emergency department with three days of confusion, severe nondescript abdominal pain, fatigue, and nausea. She denied chest pain, shortness of breath, fevers, cough, arthralgias, joint pain, skin changes, or recent viral illness. Physical exam was remarkable for a blood pressure of 223/122 mmHg and a heart rate of 130 beats per minute. There were no cardiac murmurs or rubs on auscultation, and lung sounds were clear. There was no jugular venous distention. The rest of the exam revealed no abnormalities.

**Figure 1:**
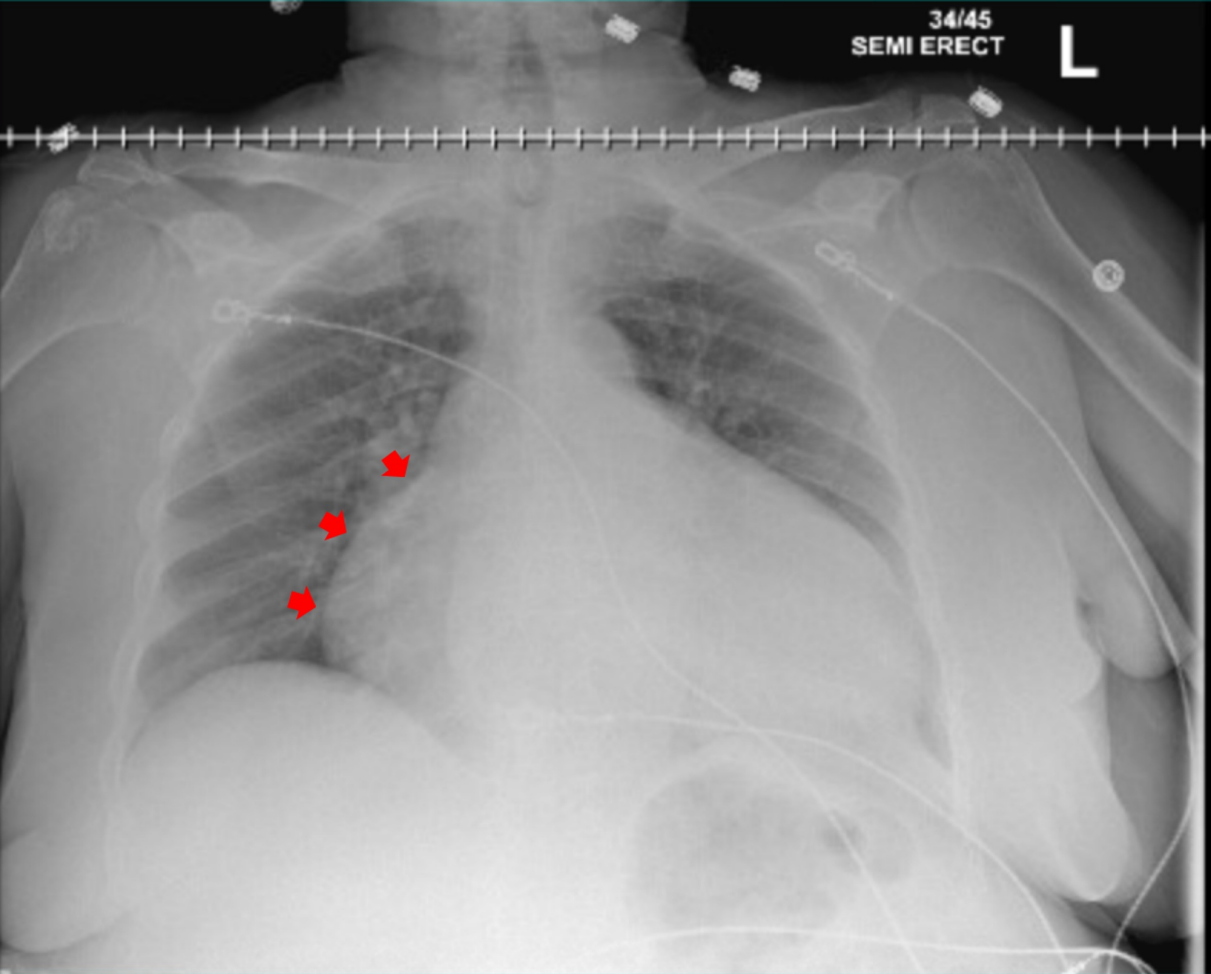
One-view upright anterior-posterior chest radiograph The image shows cardiomegaly with large cardiac silhouette, and low lung volumes. Red arrows highlight enlarged right ventricle

**Figure 2:**
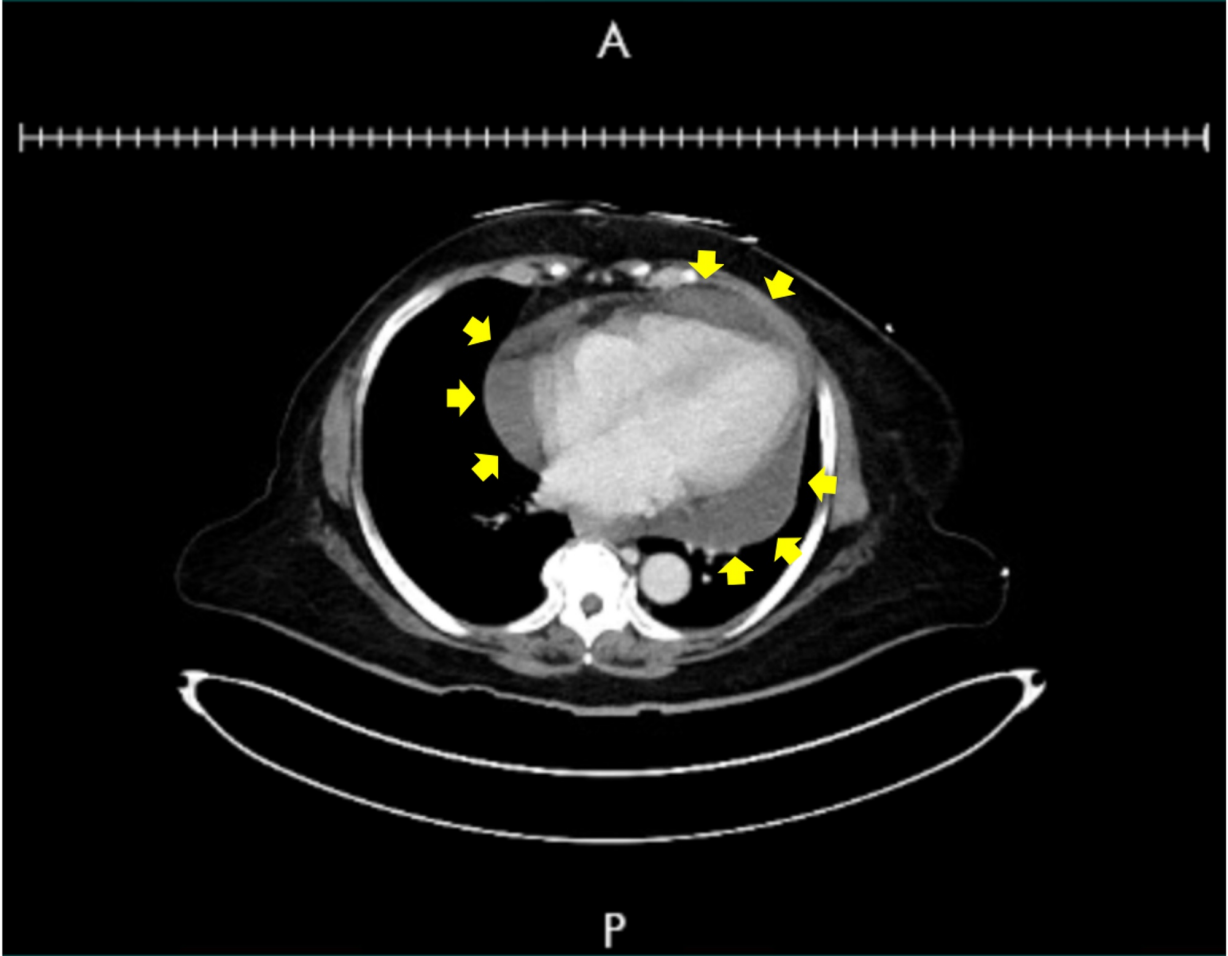
Computed tomography image The image demonstrates large pericardial effusion (yellow arrows denote outline of pericardial effusion surrounding cardiac silhouette)

**Figure 3:**
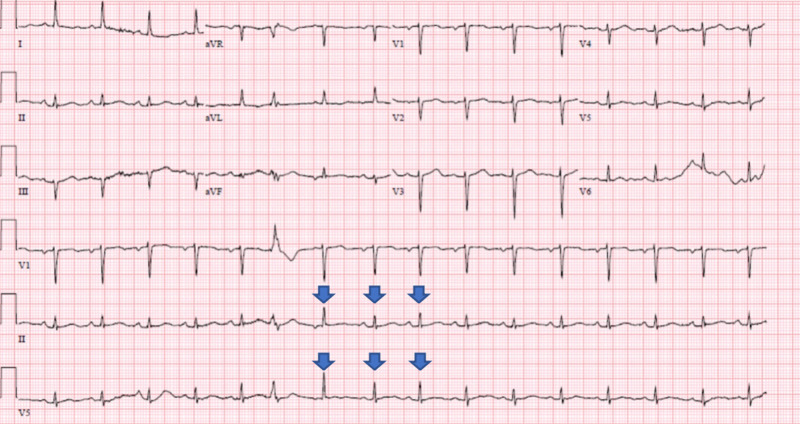
12-lead electrocardiogram The image shows low voltage QRS complexes with sinus tachycardia and electrical alternans (blue arrows demonstrate subtle beat-to-beat variation in QRS voltage amplitude); no evidence of PR segment or ST segment abnormalities

Chest radiograph (Figure 1) showed cardiomegaly, enlarged right ventricle, and low lung volumes. A CT of the abdomen was performed to evaluate her severe abdominal pain, which did not show significant intra-abdominal abnormalities but incidentally revealed a large pericardial effusion (Figure 2). Electrocardiogram (EKG) revealed low voltage QRS complexes with sinus tachycardia and electrical alternans but no evidence of PR segment or ST segment abnormalities (Figure 3). Troponin I peaked at 0.2 ng/mL, but on repeat check, it was <0.02 ng/mL (normal range = less than 0.05 ng/mL); brain natriuretic peptide was 115 pg/mL (normal range = less than 100 pg/mL), and thyroid-stimulating hormone was 0.499 mIU/L (normal range = 0.4-4.5 mIU/L). Trans-thoracic echocardiogram showed left ventricular ejection fraction (LVEF) of 65-70%, with large right-sided pericardial effusion and diastolic right ventricular collapse. On brain imaging, the patient was found to have a right parietal stroke in the setting of hypertensive emergency.

The patient developed atrial fibrillation with rapid ventricular response (RVR). In the cardiac intensive care unit, an amiodarone drip was started. A pericardiocentesis was performed and 950 cc of fluid was removed. The pericardial fluid showed high protein, 794 nucleated cells/mm^3^ with 92% lymphocytes; the cytology contained no malignant cells and culture was negative. Her anti-nuclear antibody (ANA) was positive to 1:160 (normal range = titers less than or equal to 1:40) and anti-histone antibody was positive to 1.6 (normal range = less than 1.0 units). Other rheumatologic studies were negative (rheumatoid factor, anti-double-stranded DNA, antineutrophil cytoplasmic antibodies, anti-ribonuclear protein, SSA/SSB, Scl-70, smooth muscle antibody). C3 was normal, and C4 was elevated.

A diagnosis of HILS was entertained and hydralazine was immediately discontinued. The patient was treated with colchicine 0.3 mg every other day (dosed to renal function). Her pericardial effusion was found to be resolved on repeat echocardiogram two weeks later and she was discharged home. The effusion recurred one month later despite hydralazine cessation, necessitating a pericardial window and initiation of prednisone taper, with the ultimate resolution of symptoms and effusion.

## Discussion

The differential diagnosis for pericardial effusion is broad. Given our patient’s history of breast cancer, we considered malignant effusion as a potential etiology. Other considerations included infectious etiologies, allergic mechanisms, connective tissue disorders, vasculitis, or metabolic conditions. The patient was afebrile, without leukocytosis or elevated eosinophil count, and pericardial fluid culture was negative for any infectious cause. Cytology was also negative for malignant cells. She had no recent history of primary cardiac procedures to suggest effusion as a result of a post-cardiac injury. Her renal function and thyroid function tests, as well as electrolytes, were all within normal limits, which excluded metabolic causes. Finally, rheumatologic testing revealed a positive ANA and anti-histone antibody, and thus HILS was suspected to be the most likely trigger of her pericardial effusion. It was difficult to predict whether this effusion was acute or chronic in development, as it was found incidentally in the setting of hypertensive emergency and stroke.

Pericarditis with or without pericardial effusion occurs in less than 5% of all cases of HILS [7]. Risk factors for HILS include female sex, slow hepatic acetylation, and hydralazine dose greater than 100 mg/day for longer than three months [1]. The diagnosis of HILS is based on the patient's medical history, physical examination, and laboratory findings, specifically the presence of ANA and anti-histone antibodies [9]. Discontinuation of the offending agent is the first step in management, and in severe cases, corticosteroid treatment or other immunosuppressive agents may be required [10]. Our patient developed a subacute progressive large pericardial effusion, without the characteristic symptoms of jugular venous distension on inspiration, pericarditis, hypotension, distant heart sounds, or friction rub, in the setting of chronic hydralazine therapy. Her large effusion led to atrial fibrillation with RVR, which required urgent pericardiocentesis.

## Conclusions

Large symptomatic pericardial effusion is a rare manifestation of HILS, which has been described only six times previously. Awareness of the diagnosis is important in life-threatening conditions such as cardiac tamponade. HILS should be considered in the differential diagnosis for cardiac tamponade of otherwise unclear etiology in patients taking 100 mg daily or more of hydralazine for longer than three months. Clinicians should immediately discontinue hydralazine in the process of evaluation. A temporal association between the offending drug and presenting symptoms, resolution of symptoms upon discontinuation, and a positive anti-histone antibody test can all support the diagnosis of this syndrome.
